# Single‐Microglia Transcriptomic Transition Network‐based Prediction and Real‐world Patient Data Validation Identifies Ketorolac as a Repurposable Drug for Alzheimer’s Disease

**DOI:** 10.1002/alz.089596

**Published:** 2025-01-03

**Authors:** Jielin Xu, Michael Danziger, Zhenxing Xu, Chengxi Zhang, Yadi Zhou, Yuan Hou, Ehud Karavani, Yishai Shimoni, Andrew A. Pieper, James B Leverenz, Jeffrey L. Cummings, Jianying Hu, Fei Wang, Michal Rosen‐Zvi, Feixiong Cheng

**Affiliations:** ^1^ Cleveland Clinic, Cleveland, OH USA; ^2^ IBM Research, Haifa Israel; ^3^ Institute of Artificial Intelligence for Digital Health, Weill Cornell Medicine, New York, NY USA; ^4^ Weill Cornell Medicine, New York, NY USA; ^5^ Case Western Reserve University, Cleveland, OH USA; ^6^ Cleveland Clinic Lou Ruvo Center for Brain Health, Cleveland, OH USA; ^7^ Cleveland Clinic Lou Ruvo Center for Brain Health, Las Vegas, NV USA; ^8^ IBM Research, Yorktown Heights, NY USA; ^9^ Weill Cornell Medical School, New York, NY USA

## Abstract

**Background:**

Microglia have been implicated as a key aspect of the pathology of Alzheimer’s disease (AD). However, high microglial heterogeneities, including disease‐associated microglia (DAM), tau microglia (tau‐pathology related), and neuroinflammation‐like microglia (NIM), hinder the development of microglia‐targeted treatment.

**Method:**

In this study, we integrated ∼0.7 million single‐nuclei RNA (snRNA)‐seq transcriptomes derived from AD patient frozen brain samples using a variational autoencoder. We used trajectory analysis to identify microglial subtypes across AD progression, including DAM, tau microglia, and NIM. We conducted transition network analysis to identify putative molecular drivers of microglial subtypes across varying severities of AD and disease progression under the human protein‐protein interactome network. We prioritized candidate drugs by specifically targeting transition modules using drug‐gene signature enrichment analysis and we further validated drugs using two independent real‐world patient databases (MarketScan [172 million insured individuals] and INSIGHT Clinical Research Network [15 million patients]).

**Result:**

We showed that tau microglia were significantly associated with synaptic processes. Compared to DAM, upregulated genes of NIM were significantly enriched with key immune pathways (e.g., toll‐like receptor). We identified potential AD pathobiological regulators (e.g., *SYK*, *CTSB, PRKCA*, *INPP5D*, and *ADAM10*) in transition networks between DAM and NIM. Via network‐based drug repurposing prediction by specifically targeting NIM subpopulations and real‐world patient data‐based validation, we identified that usage of ketorolac (anti‐inflammatory medicine) is significantly associated with reduced AD incidence in both MarketScan (hazard ratio [HR] = 0.81, 95% confidence interval [CI] 0.69‐0.91, p‐value = 0.002 after adjusting > 400 covariates) and INSIGHT (HR = 0.83, 95% CI 0.77‐0.92, p‐value = 0.004 after adjusting 267 covariates) patient databases.

**Conclusion:**

This study offers insights into pathobiology of AD‐relevant microglial subtypes and identifies ketorolac as a potential anti‐inflammatory treatment for AD.

